# Sensitivity of docetaxel-resistant MCF-7 breast cancer cells to microtubule-destabilizing agents including vinca alkaloids and colchicine-site binding agents

**DOI:** 10.1371/journal.pone.0182400

**Published:** 2017-08-07

**Authors:** Richard C. Wang, Xinmei Chen, Amadeo M. Parissenti, Anil A. Joy, Jack Tuszynski, David N. Brindley, Zhixiang Wang

**Affiliations:** 1 Department of Medical Genetics and Signal Transduction Research Group, Faculty of Medicine and Dentistry, University of Alberta, Edmonton, Alberta, Canada; 2 Regional Cancer Program, Sudbury Regional Hospital, Sudbury, ON, Canada; 3 Department of Oncology, Faculty of Medicine and Dentistry, University of Alberta, Edmonton, Alberta, Canada; 4 Department of Biochemistry and Signal Transduction Research Group, Faculty of Medicine and Dentistry, University of Alberta, Edmonton, Alberta, Canada; University of South Alabama Mitchell Cancer Institute, UNITED STATES

## Abstract

**Introduction:**

One of the main reasons for disease recurrence in the curative breast cancer treatment setting is the development of drug resistance. Microtubule targeted agents (MTAs) are among the most commonly used drugs for the treatment of breaset cancer and therefore overcoming taxane resistance is of primary clinical importance. Our group has previously demonstrated that the microtubule dynamics of docetaxel-resistant MCF-7_TXT_ cells are insensitivity to docetaxel due to the distinct expression profiles of β-tubulin isotypes in addition to the high expression of p-glycoprotein (ABCB1). In the present investigation we examined whether taxane-resistant breast cancer cells are more sensitive to microtubule destabilizing agents including vinca alkaloids and colchicine-site binding agents (CSBAs) than the non-resistant cells.

**Methods:**

Two isogenic MCF-7 breast cancer cell lines were selected for resistance to docetaxel (MCF-7_TXT_) and the wild type parental cell line (MCF-7_CC_) to examine if taxane-resistant breast cancer cells are sensitive to microtubule-destabilizing agents including vinca alkaloids and CSBAs. Cytotoxicity assays, immunoblotting, indirect immunofluorescence and live imaging were used to study drug resistance, apoptosis, mitotic arrest, microtubule formation, and microtubule dynamics.

**Results:**

MCF-7_TXT_ cells were demonstrated to be cross resistant to vinca alkaloids, but were more sensitive to treatment with colchicine compared to parental non-resistant MCF-7_CC_ cells. Cytotoxicity assays indicated that the IC_50_ of MCF-7_TXT_ cell to vinorelbine and vinblastine was more than 6 and 3 times higher, respectively, than that of MCF-7_CC_ cells. By contrast, the IC_50_ of MCF-7_TXT_ cell for colchincine was 4 times lower than that of MCF-7_CC_ cells. Indirect immunofluorescence showed that all MTAs induced the disorganization of microtubules and the chromatin morphology and interestingly each with a unique pattern. In terms of microtubule and chromain morphology, MCF-7_TXT_ cells were more resistant to vinorelbine and vinblastine, but more sensitive to colchicine compared to MCF-7_CC_ cells. PARP cleavage assay further demonstrated that all of the MTAs induced apoptosis of the MCF-7 cells. However, again, MCF-7_TXT_ cells were more resistant to vinorelbine and vinblastine, and more sensitive to colchicine compared to MCF-7_CC_ cells. Live imaging demonstrated that the microtubule dynamics of MCF-7_TXT_ cells were less sensitive to vinca alkaloids, and more sensitive to colchicine. MCF-7_TXT_ cells were also noted to be more sensitive to other CSBAs including 2MeOE_2_, ABT-751 and phosphorylated combretastatin A-4 (CA-4P).

**Conclusion:**

Docetaxel-resistant MCF-7_TXT_ cells have demonstrated cross-resistance to vinca alkaloids, but appear to be more sensitive to CSBAs (colchicine, 2MeOE_2_, ABT-751 and CA-4P) compared to non-resistant MCF-7_CC_ cells. Taken together these results suggest that CSBAs should be evaluated further in the treatment of taxane resistant breast cancer.

## Introduction

Breast cancer (BC) is the most common type of cancer in women accounting for approximately one third of all cancers. The incidence of BC continues to increase annually, with more than one million reported new cases currently diagnosed each year worldwide [1–3]. Among these cases, 20–30% present with metastatic or locally advanced disease, and another 30% will develop recurrent or metastatic disease [3].

Metastatic breast cancer (MBC) is among the leading causes of cancer mortality [4, 5]. Front line chemotherapy regimens for treating MBC typically include a taxane such as docetaxel, paclitaxel or nab-paclitaxel, either alone or in combination [5–9]. Despite initial responses, metastatic disease will eventually progress while on treatment primarily due to the development of drug resistance [10]. Subsequent chemotherapeutic agents used clinically for taxane-resistant BC include anthracyclines, antimetabolites (e.g. capecitabine, gemcitabine), vinca alkaloids (e.g. vinorelbine) and halichondrin class agents (e.g. eribulin), however, with limited progressive success.

To date, microtubule-targeting agents (MTAs) remain one the most reliable classes of antineoplastic drugs in the treatment of BC [11]. Microtubules are hollow cylindrical cores composed of α- and β-tubulin heterodimers [12, 13]. The polymerization and depolymerisation dynamics of microtubules are of key importance in cellular function, especially in spindle formation during mitosis [13, 14]. MTAs disrupt microtubule dynamics, leading to abnormal mitotic spindles, chromosome misalignment and the perpetual activation of the spindle assembly checkpoint (SAC). MTAs can be divided into two categories: microtubule-destabilizing agents such as vinca alkaloids and colchicine-site binding agents, and microtubule-stabilizing agents such as taxanes [15].

Taxanes function primarily by interfering with spindle microtubule dynamics. Stabilization of microtubules by taxane binding prevents the normal formation of mitotic spindles and their disassembly [16]. This leads to chronic activation of the SAC, which in turn *leads* to mitotic arrest [17] and eventual cell death [18]. The cellular target for taxanes are the taxane site on β-tubulin facing the lumen of a microtubule and stabilizing the M-loop of tubulin which prevents disassembly [12, 19]. The taxane-binding site on microtubules is only present in assembled tubulin [20].

Vinca alkaloids (e.g. vincristine, vinblastine, and vinorelbine), target both unpolymerized tubulin dimers and microtubules by binding to the vinca domain of β-tubulin. CSBAs (e.g. 2-MeOE_2_, STX140 and colchicine), bind to the colchicine domain and only bind to soluble dimers, which are then unable to incorporate into microtubules due to an induced conformational change of the dimer upon binding [11]. While the mode of action may be different from the microtubule stabilizers, it is the SAC-dependent mitotic delay that enhances the vulnerability of cells to SAC-induced cell death [15, 21].

We have recently demonstrated that multiple mechanisms are involved in the resistance to taxanes in MCF-7_TXT_ cells that was selected for docetaxel resistance [22, 23]. We have shown that the microtubule dynamics of docetaxel-resistant MCF-7_TXT_ cells are insensitive to the docetaxel treatment, which may partially explain why docetaxel is less effective in inducing mitotic (M-phase) arrest and apoptosis in MCF-7_TXT_ cells when compared with the parental MCF-7_CC_ cells [23]. It is logical to hypothesize that the microtubule dynamics that are resistant to microtubule stabilization by drugs such as taxanes could be more sensitive to microtubule destabilizing agents such as vinca alkaloids and CSBAs. Thus, taxane-resistance could possibly be overcome with microtubule destabilizing agents. Reports have noted increased β2-, β3- and β-4 tubulin expression levels being associated with taxane resistance, decreased expression of β3-tubulin in vinca-resistant cell lines, together with increased microtubule stability [24]. Moreover, β3-tubulin overexpression has also been reported to confer resistance to paclitaxel and vinorelbine, but with no effect to the resistance to colchicine-site binding agents [25]. Lastly, STX140, but not paclitaxel, inhibits mammary tumour initiation and progression in transgenic mice [26].

In this work, by using various methods we have tested the sensitivity of MCF-7_TXT_ cells to microtubule-destabilizing agents, including vinca alkaloids and CSBAs. We found that docetaxel-resistant MCF-7TXT cells were cross-resistant to vinca alkaloids, however, they were more sensitive to CSBAs including colchicine, 2MeOE2, ABT-751 and CA-4P than the non-resistant MCF-7CC cells.

## Materials and methods

### Cell culture and treatment

The cell lines that were used in this study include MCF-7 breast cancer cells selected for resistance to docetaxel (MCF-7_TXT_), and the non-resistant cell line “selected” by propagation of MCF-7 cells to the same passage number in the absence of drug (MCF-7_CC_), as we previously described [22]. All cells were grown at 37°C in Dulbecco's modified Eagle's medium containing 10% FBS supplemented with non-essential amino acids and they were maintained in a 5% CO_2_ atmosphere. MCF-7_TXT_ cells were cultured with additional taxel of 5 nM to maintain the resistance to docetaxel. Howevr, this 5nM docetaxel was removed when the cells were treated for experiments.

### Antibody and chemicals

Mouse monoclonal anti- antibody was purchased from Santa Cruz Biotechnology, Inc. (Santa Cruz, CA). Rabbit anti-tubulin α antibody was from Sigma-Aldrich (Oakville, Ontario). All secondary antibodies conjugated with FITC and TRITC were obtained from Life Technologies Inc (Burlington, ON, Canada). Mammalian Protein Extraction Reagent (M-Per) was purchased from Thermo Fisher Scientific Inc. (Rockford, IL). Vybrant MTT Cell Proliferation Assay Kit and GFP tagged α-tubulin with the CellLight^®^ Reagents BacMam 2.0 were from Invitrogen (Grand Island, NY). Unless otherwise specified, all other chemicals were purchased from Sigma-Aldrich.

### Cytotoxicity assay

Cells were plated onto 96-well plates, 10,000 cell/well for each MCF-7 cell line. The culture medium was replaced 48 h later with fresh medium containing various concentrations of drugs for 48 h. The percentages of viable cells were then determined by the conversion of the water soluble MTT (3-(4,5-dimethylthiazol-2yl)-2,5-diphenyltetrazolium bromide) to an insoluble formazan, relative to drug free controls, using the Vybrant MTT Cell Proliferation Assay Kit (Invitrogen, Grand Island, NY). All cytotoxicity data shown are the means of at least three independent experiments. Similar experiment were performed with drug treatment for 72 h.

### Apoptosis assay

Cell apoptosis was determined by two different methods: chromatin condensation/fragmentation and the cleavage of PARP.

Forchromatin condensation/fragmentation, cells on cover slips were treated with various concentrations of drugs for 24 h. DNA was stained with 300 nM DAPI for 5 min. Chromatin condensation and nuclear morphology were examined using a Delta Vision microscopic system with Delta Vision SoftWoRx software to deconvolve the images. For each experiment, we counted 100 cells and identified the number of apoptoic cells to calculate the percentage of apoptosis. Each value was the mean of three experiments. so that 300 cells were examined for the quantification.

PARP cleavage was determined by immunoblotting. Briefly, protein lysates from two MCF-7 cell lines treated or not treated with various concentrations of drugs were obtained by lysing cells with M-PER Mammalian Protein Extraction Reagent contianing a protease inhibitor cocktail. PARP was detected by immunoblotting and PARP cleavage was quantified by densitometry. The arbitrary intensity of each cleave PARP band was measured and normalized against the background, which was set as zero. Each value was the mean of three experiments.

### Indirect immunofluorescence

Indirect immunofluorescence was described previously [27]. For the staining of ABC proteins, cells were either treated or not treated with docetaxel at indicated concentrations for 24 h. After fixation with methanol at -20°C, cells were incubated with antibody against α-tubulin, followed by FITC-conjugated secondary antibodies. Nuclei were then counter stained with DAPI. The images were obtained using a Delta Vision microscopic system as above.

### Live imaging

Live imaging was used to study docetaxel induced M-phase arrest and microtubule dynamics. As described previously [27], MCF-7_CC_ and MCF-7_TXT_ cells were cultured on 35mm poly-L-lysine-coated coverslips (Fisher) overnight. To assay docetaxel-induced M-phase arrest, cells were incubated with DMEM containing 250 ng/ml Hoechst 33342 (Calbiochem) for 10 min to stain DNA. Then, coverslips were mounted on a sample holder and incubated with DMEM without phenol red, supplemented with 10% FBS with or without docetaxel. To assay microtubule dynamics, cells were transfected with GFP tagged α-tubulin with the CellLight^®^ Reagents BacMam 2.0 (Invitrogen, Grand Island, NY) for 24 h according to the Manufacturer’s instructions. Coverslips were then mounted on a sample holder and incubated with DMEM without phenol red, supplemented with 10% FBS either with or without docetaxel as indicated for 1 h.

Experiments were performed in a chamber maintained at 37°C and 5% CO_2_. Fluorescence images were acquired at the indicated times using a Delta Vision microscopic system. Delta Vision SoftWoRx software was used to deconvolve images and generate movies.

## Results

### Sensitivity of taxane-resistant MCF-7_TXT_ cells to vinca alkaloids and colchicine

**Fig 1:**
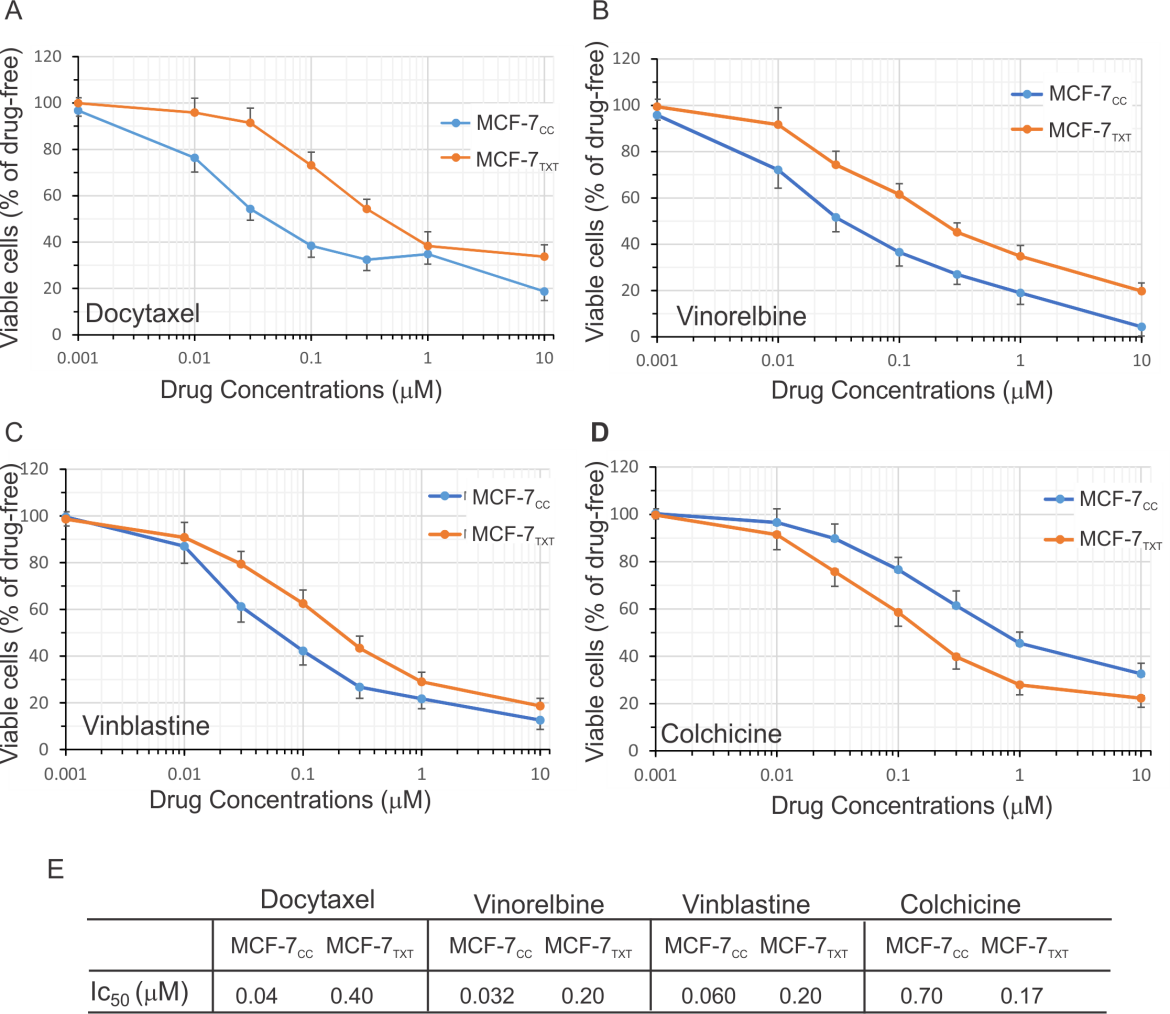
Dose-response curve to drug treatment. Cytotoxicity of docetaxel, vinorelbine, vinblastine and colchicine at various concentrations for MCF-7_CC_ and MCF-7_TXT_ cells was determined following a 24 h treatment with (A) docetaxel; (B) vinorelbine. (C) Treatment with vinblastine. (D) Treatment with colchicine. (E) IC_50_s calculated from A-D. The means of at least three independent experiments are plotted.Error bars are the standard errors.

The sensitivity to various drugs was determined by using a cytotoxicity assay based on the Vybrant MTT Cell Proliferation Assay following drug treatment for 48 h (Fig 1). The IC_50_ values for various drugs were determined from the dose-response curve of this cytotoxicity assay (Fig 1E). MCF-7_TXT_ cells were more than 6 times more resistant to vinorelbine than MCF-7_CC_ cells and 6 times more resistance to vinblastine than MCF-7_CC_ cells; however, MCF-7_TXT_ cells showed no resistance to colchince. MCF-7_TXT_ cells appeared to be more sensitive to colchicine than MCF-7_CC_ cells. These results indicate that while both vinca alkaloids and colchicine destabilize microtubules, they have different effects on taxane-resistant breast cancer cells.

### Effects of vinca alkaloids and colchicine on chromatin morphology, apoptosis and microtubule formation

**Fig 2:**
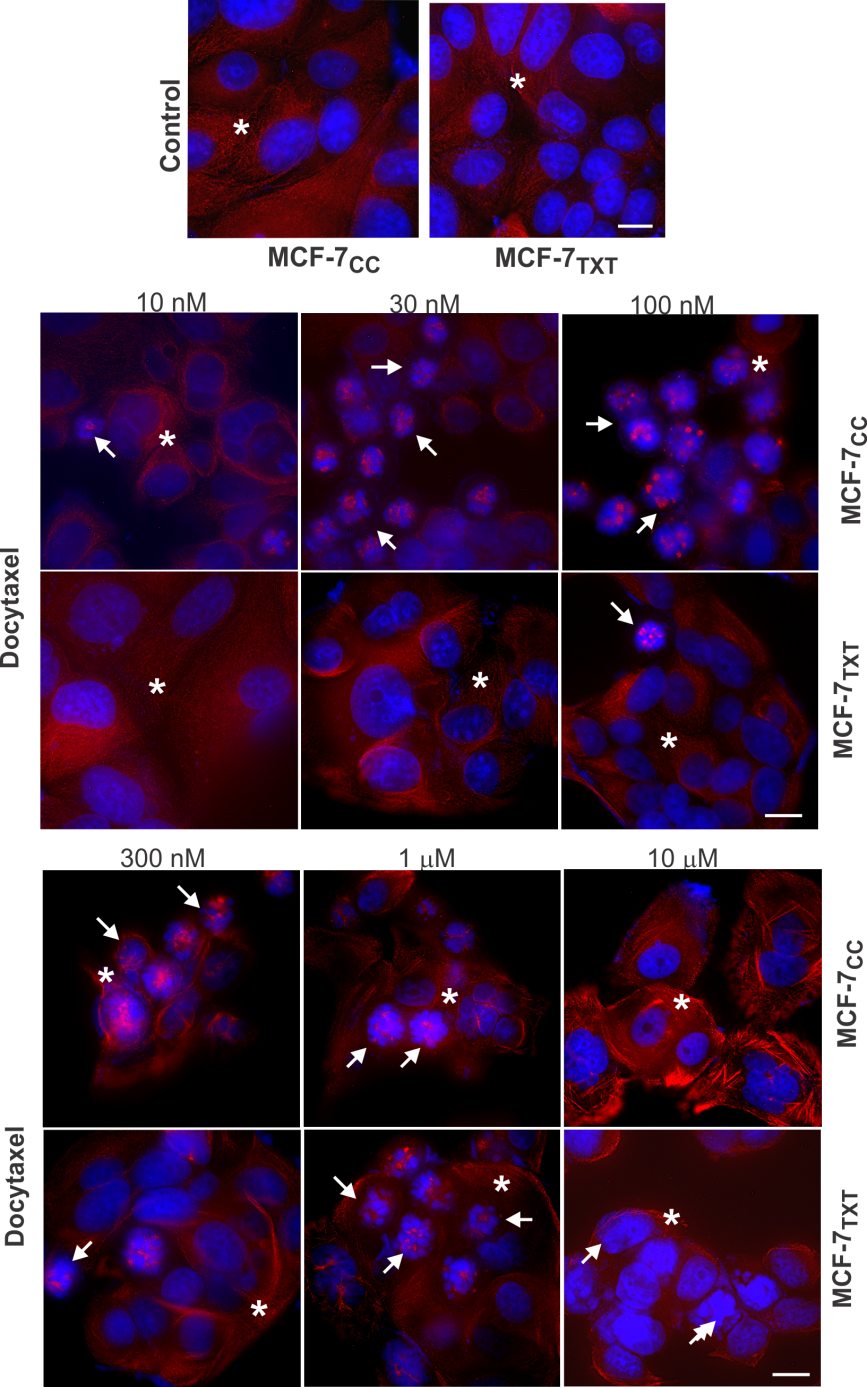
The effects of docetaxel on the microtubule formation, M-phase arrest and cell apoptosis in the selected MCF-7 cell lines. MCF-7_CC_ and MCF-7_TXT_ cells were seeded on coverslip and treated with docetaxel of indicated concentrations for 24 hours. The microtubule formation, M-phase arrest and cell apoptosis were determined by fluorescence microscopy. The red indicates the α-tubulin and the blue is the DAPI stain for DNA. Size bar: 10 μm.

**Fig 3:**
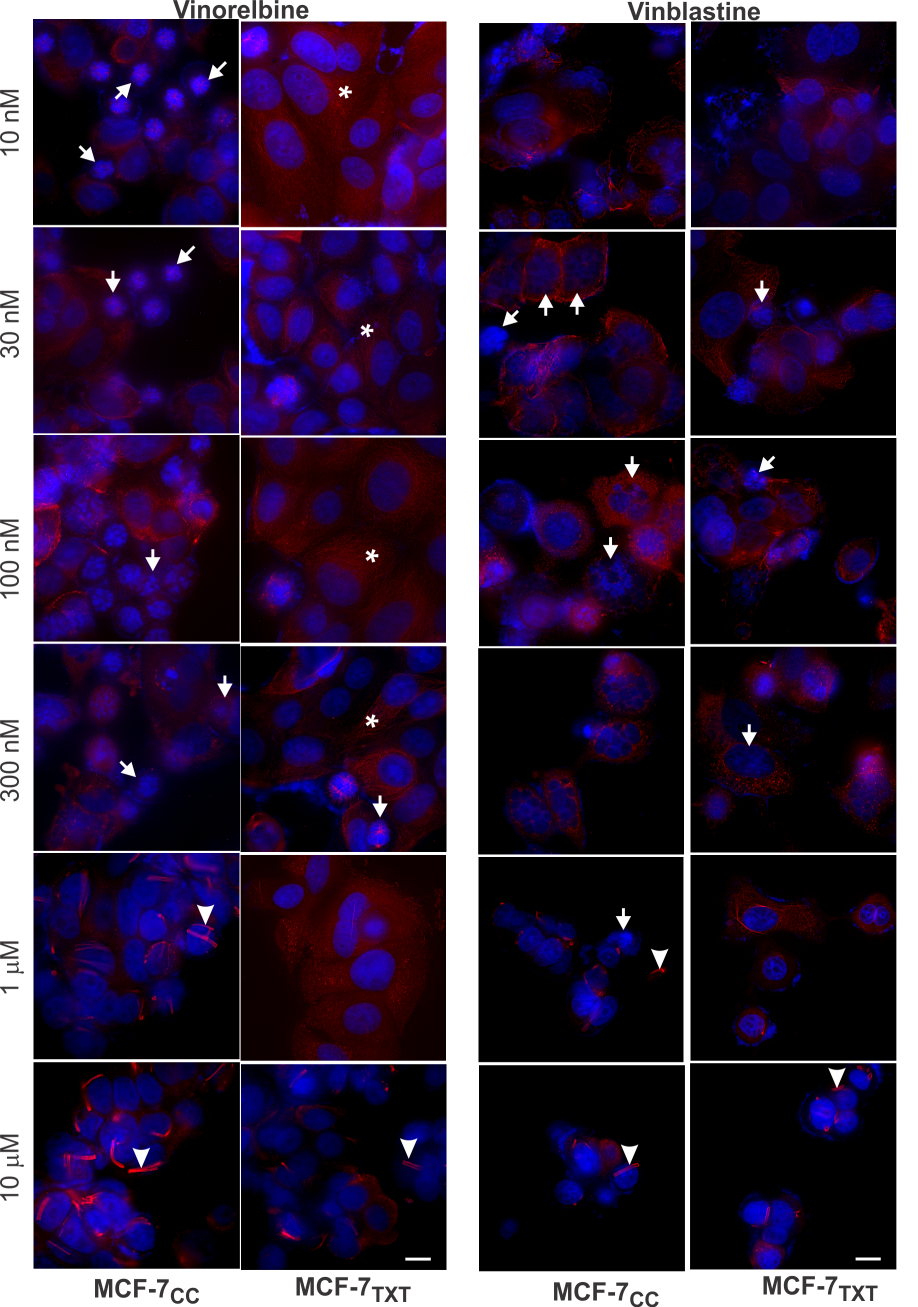
The effects of vinca alkaloids on the microtubule formation, M-phase arrest and cell apoptosis in the selected MCF-7 cell lines. MCF-7_CC_ and MCF-7_TXT_ cells were seeded on coverslip and treated with vinorelbine or vinblastine of indicated concentrations for 24 hours. The microtubule formation, M-phase arrest and cell apoptosis were determined by fluorescence microscopy. The red indicates the α-tubulin and the blue is the DAPI stain for DNA. Size bar: 10 μm.

**Fig 4:**
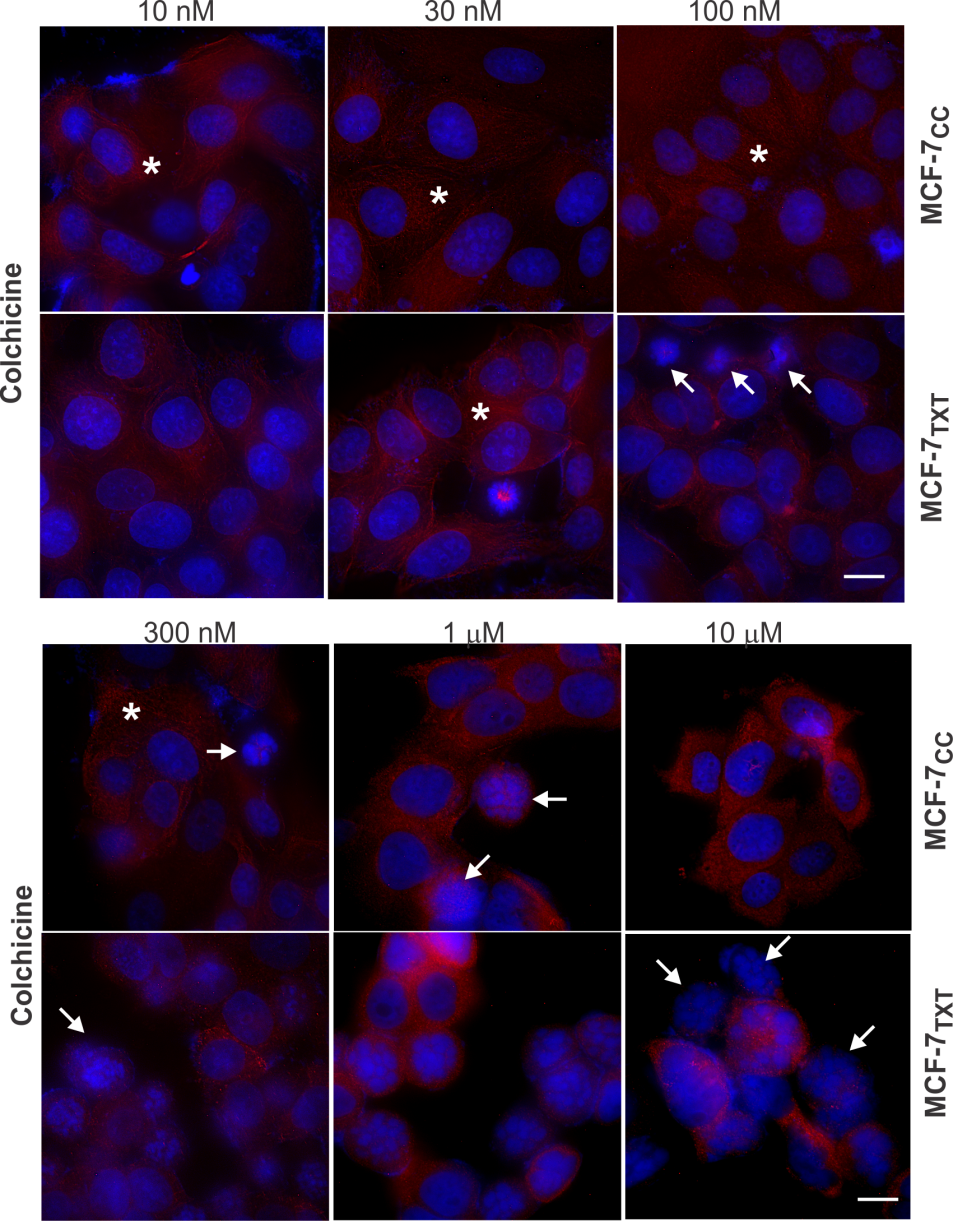
The effects of colchicine on the microtubule formation, M-phase arrest and cell apoptosis on the selected MCF-7 cell lines. MCF-7_CC_ and MCF-7_TXT_ cells were seeded on coverslip and treated with colchicine of indicated concentrations for 24 hours. The microtubule formation, M-phase arrest and cell apoptosis were determined by fluorescence microscopy. The red indicates the α-tubulin and the blue is the DAPI stain for DNA. Size bar: 10 μm.

**Fig 5:**
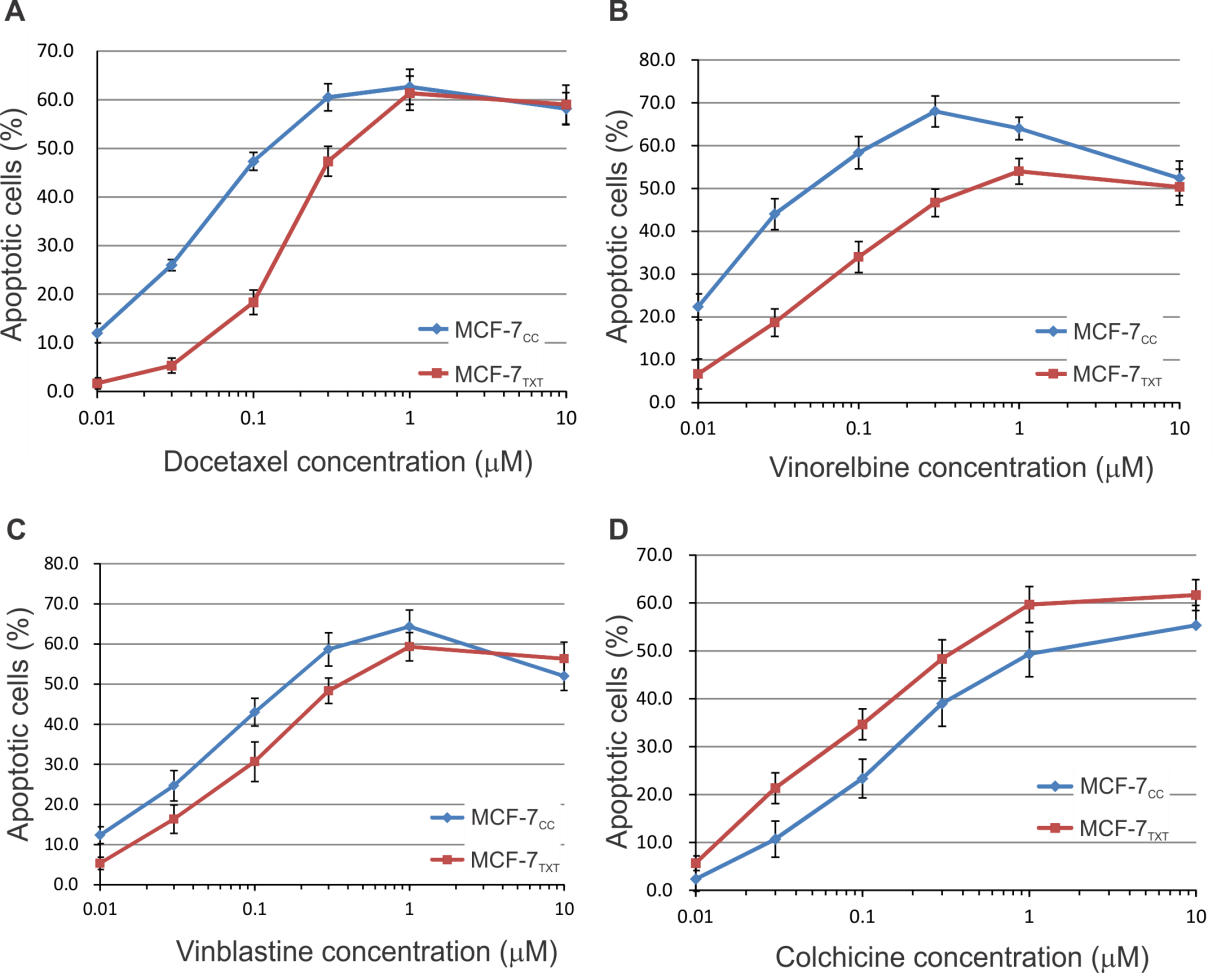
The effects of docetaxel, vinorelbine, vinblastine and colchicine on the apoptosis of MCF-7_CC_ and MCF-7_TXT_ cells. The apoptotic cells were determined by chromatin condensation and nuclear morphology as revealed by DAPI stain in Figs 2–4. Quantification of cell apoptosis was expressed as the percentage of the apoptotic cells of the total cells. For each data, 300 cells from at least three independent experiments were examined. Error bars are the standard errors.

MTAs kill cells by either stabilizing or destabilizing microtubules, which eventually causes mitotic arrest and cellular apoptosis. We therefore examined the effects of vinca alkaloids and colchicine using fluorescence microscopy by determining chromatin morphology with DAPI staining (blue), apoptosis by nuclear condensation with DAPI and microtubule formation with tubulin stain (red) (Figs 2–4). Cell apoptosis was quantitated by calculating the percentage of apoptotic cells at each given drug concentration (Fig 5).

As expected from our previous report [23], docetaxel induced much more pronounced cell apoptosis in MCF-7_CC_ cells than that in MCF-7_TXT_ cells at <2 μM (Figs 2 and 5). Docetaxel began to induce apoptosis at 10 nM in MCF-7_CC_ cells (10%), but a similar level of apoptosis in MCF-7_TXT_ cell only occured when 100 nM docetaxel was used. Maximum apoptosis (~60%) in MCF-7_CC_ cells was induced by 500 nM docetaxel, whereas a similar level of apoptosis in MCF-7_TXT_ cell required 2 μM docetaxel. However, ≤2 μM, docetaxel caused a similar level of apoptosis in both cell lines. These results may indicate that the resistance of MCF-7_TXT_ cells to docetaxel is due to their resistance to docetaxel-induced apoptosis.

The effects of three microtubule-destabilizing agents on the formation of microtubules were very different from those induced by docetaxel. Low concentrations (10 nM to 100 nM) of vinorelbine destabilized the microtubules in the non-resistant MCF-7_CC_ cells (Fig 3). The microtubule fibers were much reduced at 10 nM and completely disappeared at 100 nM. Vinorelbine at 1–10 μM caused the crystallization of soluble tubulins. Similar effects were observed for the resistant cells, but a much higher vinorelbine concentration was needed. Microtubule fibers disappeared completely at 1 μM, which is ten times higher compared to MCF-7_CC_ cells. Crystallization of soluble tubulin started at 10 μM, which is also ten times higher. While the effects of vinorelbine on the microtubule formation were opposite to that of docetaxel, both vinorelbine and docetaxel had the similar effects on the chromatin condensation and fragmentation. The morphology of the condensed chromatin and the pattern of the fragmentation were very similar following the treatment of either vinorelbine or docetaxel. Vinorelbine induced the significant chromatin condensation and fragmentation at 10 nM for the non-resistant cells, but only induced weak chromatin condensation at 300 nM for the resistant cells. The effects on apoptosis, as calculated based on chromatin morphology, showed that vinorelbine induced higher apoptosis at lower concentrations in MCF-7_CC_ cells than in MCF-7_TXT_ cells (Fig 5B). These results further indicate that the docetaxel-resistant MCF-7_TXT_ cells were also cross-resistant to vinorelbine.

Vinblastine had a very similar effects on microtubule morphology when compared with vinorelbine (Figs 3& 5C). For the non-resistant MCF-7_CC_ cells, vinorelbine significantly destabilized the microtubules at 10 nM to 30 nM,. The microtubule fibers were much reduced and disorganized at 10 nM. At 100 nM the microtubules completely disappeared and the tubulin appeared as separated dots. At 1–10 μM, vinblastine causes crystallization of soluble tubulins. Similar effects were observed for the resistant cells, but a relatively higher vinblastine concentration was needed. The microtubule fibers disappeared completely at 300 nM and crystallization of soluble tubulin started at 10 μM (Fig 3).

However, the effects of vinblastine on the morphology of cell nuclei were quite different from vinorelbine. Vinblastine induced significant fragmentation of nuclei and induced multiple small round condensed chromatin rings (Fig 3). This difference in nucleus morphology suggests that the mechanism of action of vinblastine may not be exactly the same as vinorelbine. The effects on apoptosis, as calculated based on chromatin morphology, showed that vinblastine also induced strong apoptosis in both cell lines. The docetaxel resistant MCF-7_TXT_ cells were also cross-resistant to vinblastine in terms of apoptosis, but to a lesser degree when compared with vinorelbine.

It is significant that the resistant MCF-7_TXT_ cells were more sensitive to colchicine when compared with non-resistant MCF-7_CC_ cells (Figs 4& 5D), which is consistent with our cytotoxicity assay (Fig 1). For the non-resistant MCF-7_CC_ cells, microtubules had completely disappeared at 1 μM colchicine and the chromatin condensation began at 300 nM (Fig 4). However, for resistant MCF-7_TXT_ cells, the microtubules had completely disappeared at 100 nM colchicine and chromatin condensation began at 30 nM. From the enlarged images, it can be seen that tubulin was stained as many small dots, rather than a fiber, which is consistent with the function of colchicine as a microtubule destabilizer. Colchicine did not cause the crystallization of tubulins even at 10 μM, which distinguishes it from vinca alkaloids including vinorelbine and vinblastine. Moreover, the morphology of the condensed chromatin and fragmented nucleus induced by colchicine was also different from that induced by vinorelbine and vinblastine.

The effects on apoptosis, as calculated based on chromatin morphology, also showed that colchicine was more effective at inducing cell apoptosis in MCF-7_TXT_ cells than that in MCF-7_CC_ cells (Figs 4& 5D), which is consistent with our cell toxicity assay (Fig 1). Colchicine very strongly induced apoptosis for both cell lines, however, colchicine was five times more effective in inducing apoptosis in docetaxel resistant MCF-7_TXT_ cells than the non-resistant MCF-7_CC_ cells (Fig 5D).

**Fig 6:**
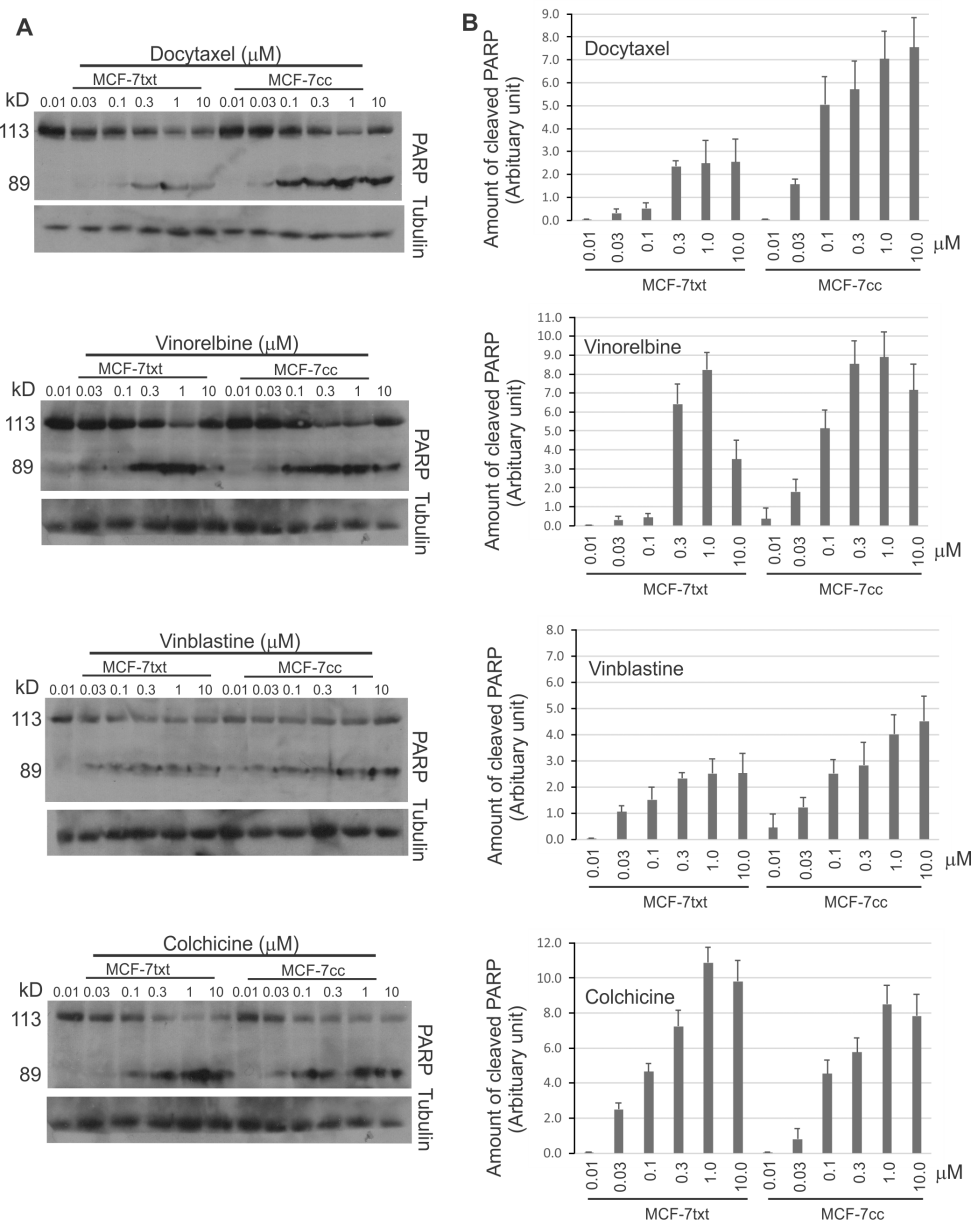
The effects of docetaxel, vinorelbine, vinblastine and colchicine on the cleavage of PARP in selected MCF-7 cell lines. MCF-7_CC_ and MCF-7_TXT_ cells were treated with various drugs at indicated concentration for 24 h. (A) The cleavage of PARP in MCF-7_CC_ and MCF-7_TXT_ cells was determined by immunoblotting with antibody to PARP. (B) Quantification of the results from three independent experiments as described in panel A. The intensity of the bands of various tubulin proteins was normalized against the intensity of the actin loading. Error bars are the standard errors. Statistical analysis with paired t-Test indicated that the difference between MCF-_7CC_ and MCF-7_TXT_ cells was statistically significant with p<0.01 for all four drugs including docetaxel, vinorelbine, vinblastine, and cochicine.

Lastly we examined the effects of these drugs on cell apotpotsis by measuring PARP cleavage (Fig 6). Docetaxel, vinorelbine, vinblastine, and cochicine induced PARP cleavage in a dose-dependent manner. MCF-7_TXT_ cells were more resistant to docetaxel in terms of PARP cleavage than MCF-7_CC_ cells (Fig 6). MCF-7_TXT_ cells were also cross-resistant to vinorelbine and vinblastine, whereas they were more sensitive to cochicine than MCF-7_CC_ cells. The difference between MCF-7_CC_ and MCF-7_TXT_ cells was statistically significant (p<0.01) for all four drugs. The PARP cleavage results were clearly consistent with our early results in this paper obtained by examining chromatin morphology, which indicates strongly that docetaxel resistant MCF-7_TXT_ cells are more sensitive to colchicine-linduced apoptosis.

Together, these results demonstrate that all of the MTAs induced the disorganization of microtubules and the chromatin morphology. Docetaxel-resistant MCF-7_TXT_ cells were cross-resistant to both vinorelbine and vinblastine. However, the docetaxel-resistant MCF-7_TXT_ cells were more sensitive to colchicine than the non-resistant MCF-7_CC_ cells.

### Live imaging of the effects of vinca alkaloids and colchicine on microtubule formation

**Fig 7:**
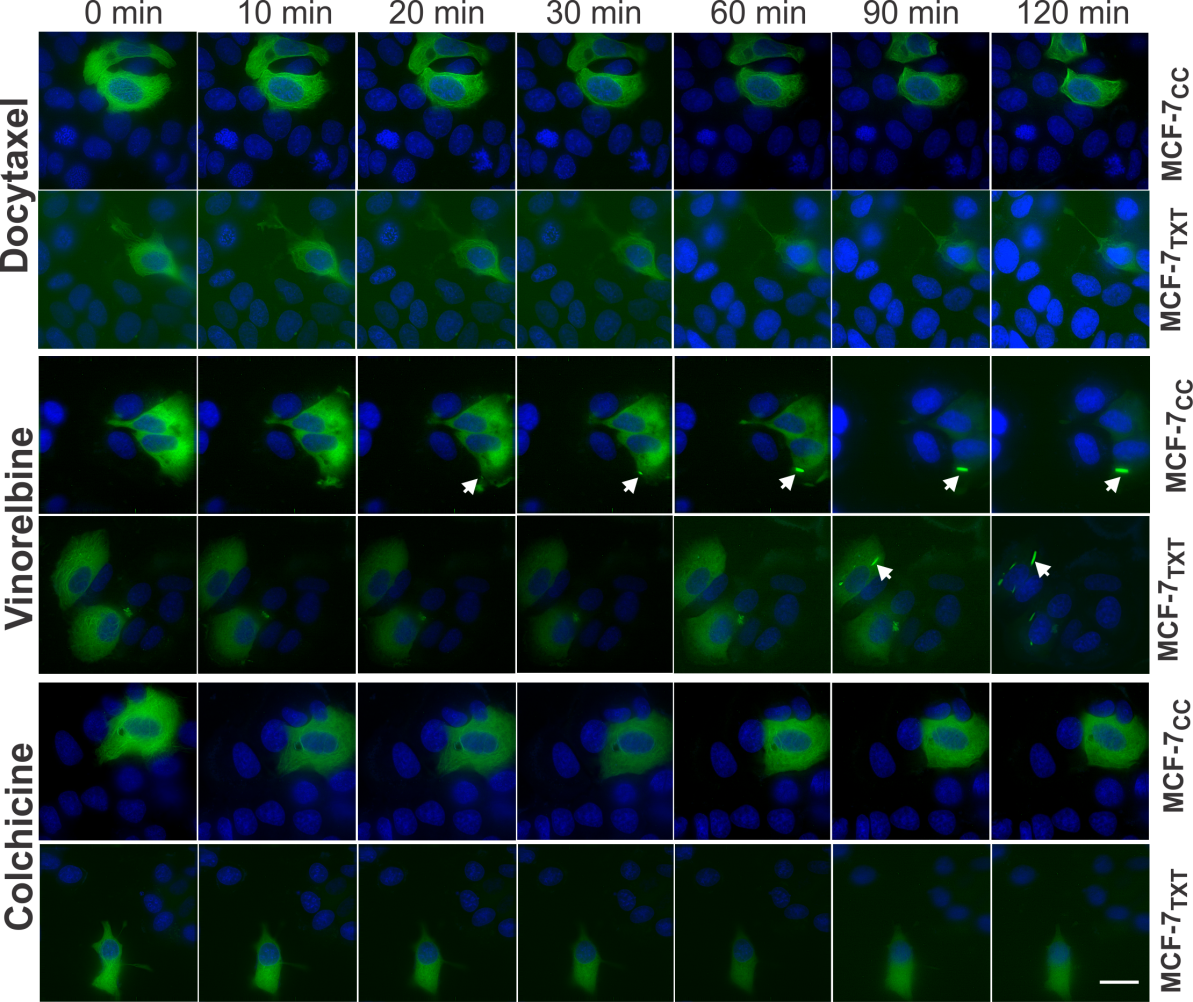
The effects of MTAs (docetaxel, vinorelbine and colchicine) on the microtubule dynamics of MCF-7_TXT_ an MCF-7_CC_ cells. The Live imaging was performed following the transfection of the cells with GFP-tagged α-tubulin for 24 h. Cells were incubated with various MTAs at 2μM for 2 h. Live images of microtubule dynamics of MCF-7_CC_ and MCF-7_TXT_ cells were recorded every 3 min. Selected images from the live imaging (S1–S6 Videos) of MCF-7_CC_ and MCF-7_TXT_ cells were shown. Arrow indicates the crystallization of tubulins. Size bar, 10 μm.

The fluorescence results described above were obtained after the cells had been treated with the drugs for 24 h. We next used live imaging (time-lapse microscopy) to show the time course. We transiently expressed GFP-tubulin in MCF-7TXT and MCF-7CC cells for 24 h. The effects of various drugs on the microtubule dynamics and morphology were followed by time-lapse fluorescence microscopy for up to 2 h (Fig 7 and S1–S6 Videos). Since we first had to transfer the coverslip (with cells) into the sample holder with the medium containing the drugs, we only recorded the images after 5 min due to the time needed for localizing the cells and for setting up the recording parameters.

To observe the effects in a relatively short time, we used high drug concentrations (10 μM). As we showed early by indirect immunofluorescence, 10 μM docetaxel stimulated the formation of thick microtubule bundles (Fig 2). The effect of docetaxel in the non-resistant cell was much stronger than that in resistant cells (Fig 7A, S1 and S2 Videos). Microtubule fibers were already much more visible and thicker in MCF-7_CC_ cells after 5 min of docetaxel treatment than in MCF-7_TXT_ cells. At 90 and 120 min, microtubule bundles were clearly visible in MCF-7_CC_ cells, but no microtubule bundles were formed in MCF-7_TXT_ cells. These results show that the microtubule system of MCF-7_TXT_ cells was not sensitive to docetaxel when compared with MCF-7_CC_ cells.

We next examined the effects of vinorelbine. Ten μM vinorelbine destabilized microtubule fibres completely and induced the crystallization of soluble tubulins (Fig 3). Live imaging of the non resistant MCF-7CC cells showed that vinorelbine destabilized the microtubules much more quickly (Fig 7B and S3 and S4 Videos). At 5 min following vinorelbine treatment, no organized microtubules were visible. However, for the resistant MCF-7_TXT_ cells, it took 20 min for vinorelbine to completely destabilize the microtubules. Vinorelbine-induced crystallization of soluble tubulin was first observed at 20 min in MCF-7_CC_ cells, but this took 90 min in MCF-7_TXT_ cells (Fig 7B). This result is consistent with our other results that docetaxel resistant MCF-7_TXT_ cells are less sensitive to vinorelbine in terms of microtubule dynamics.

Finally, we examined the effects colchicine, which at 10 μM induced the complete disappearance of microtubules as showed above (Fig 4). Live imaging further indicated that colchicine reduced the presence of microtubules immediately following the drug treatment (Fig7C and S5 and S6 Videos). At 20 min, most microtubules had already disappeared and at 60 min there were no visible microtubules in docetaxel-resistant MCF-7_TXT_ cells. However, in the non-resistant MCF-7_CC_ cells, the effects of colchicine were much less visible. Colchicine only reduce the presence of microtubules slowly and some microtubules were still visible even at 120 min (Fig 7C). Thus, these results further showed that docetaxel-resistant cells were more sensitive to colchicine in terms of microtubule dynamics, which is consistent with our results in Fig 1 that MCF-7_TXT_ cells had a lower IC_50_ than MCF-7_CC_ cells.

### Effects of other cochicine site binding agents (CSBAs)

Our above results so far indicated that docetaxel-resistant MCF-7_TXT_ cells were more sensitive to colchicine in terms cell cytotoxicity, apoptosis and microtubule formation/dynamics. We, therefore, examined whether colchicine could potentially be used to overcome taxane resistance using MCF-7_TXT_ cell lines using other CSBAs including 2MeOE_2_, ABT-751 and phosphorylated combretastatin A-4 (CA-4P).

**Fig 8:**
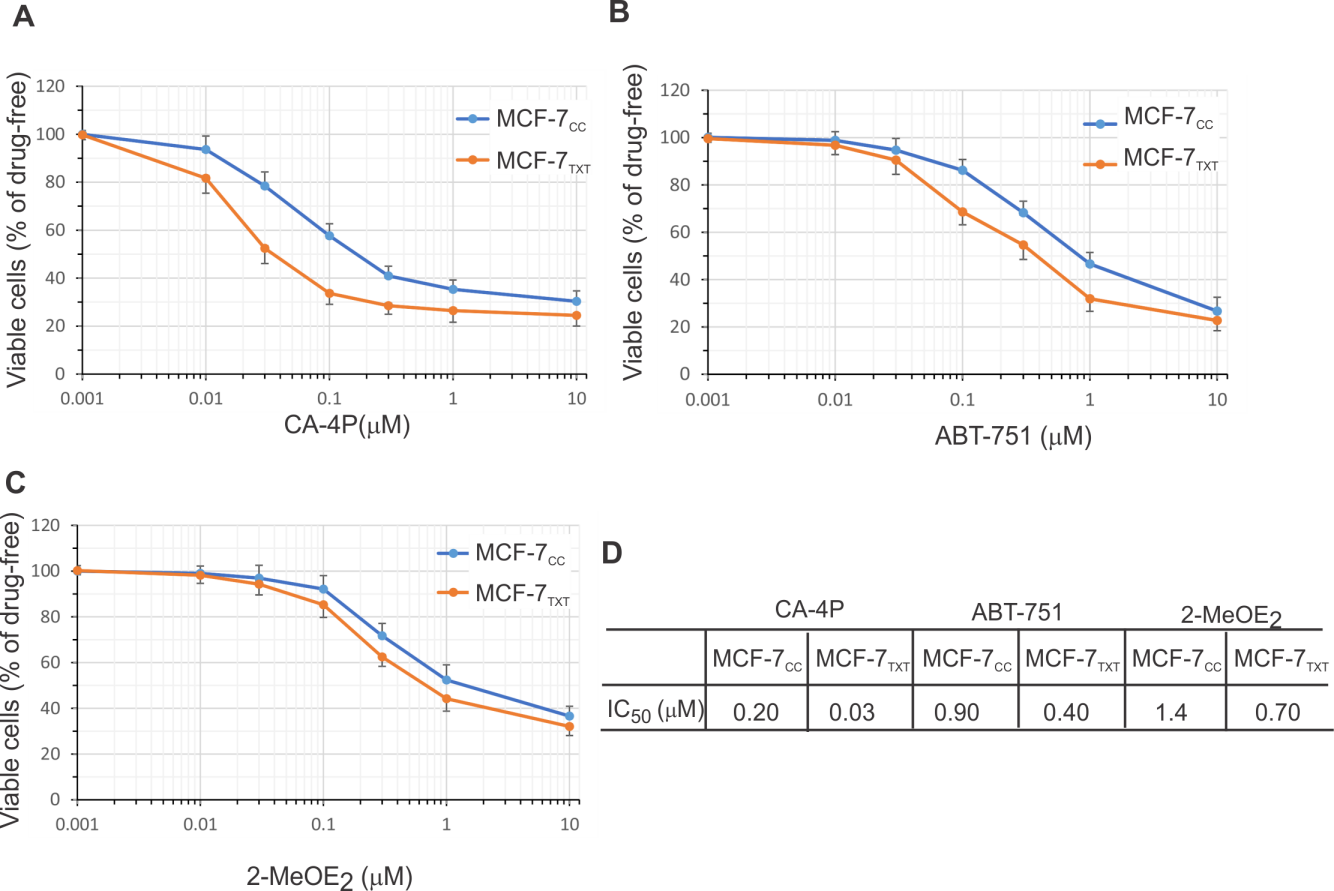
Dose-response curve to CSBA treatment. Cytotoxicity of CA-4P, ABT-751, and 2MeOE_2_ for MCF-7_CC_ and MCF-7_TXT_ cells was determined at various concentrations following 24 h drug treatment. Treatments with CA-4P (A), ABT-751 (B), 2MeOE_2_ (C) are shown IC_50_s calculated from A-C are given in (D). The means of at least three independent experiments are plotted. The error bars are the standard errors.

We first evaluated the sensitivity of taxane-resistant MCF-7_TXT_ cells to these CSBAs by using a cytotoxicity assay based on the Vybrant MTT Cell Proliferation Assay following drug treatment for 48 h as described above (Fig 8). The IC_50_ for various drugs were determined for the two cell lines from the dose-response curve from this cytotoxicity assay (Fig 8D). Docetaxel-resistant MCF-7_TXT_ cells were more sensitive to all three CSBAs than the non-resistant MCF-7_CC_ cells. However, the efficacies of these three CSBAs were very different. The IC_50_ of CA-4P was much lower than that of colchicine, indicating the high toxicity of CA-4P to these breast cancer cells. MCF-7_TXT_ cells were nearly 6-times more sensitive to CA-4P than MCF-7_CC_ cells. Both 2MeOE_2_ and ABT-751 were ineffective in causing cytotoxicity in MCF-7_CC_ and MCF-7_TXT_ cells. The IC_50_s of 2MeOE_2_ and ABT-751 were in the μM range (Fig 8D). However, MCF-7_TXT_ cells were still more sensitive to both 2MeOE_2_ and ABT-751 than MCF-7_CC_ cells, although to a lesser degree.

**Fig 9:**
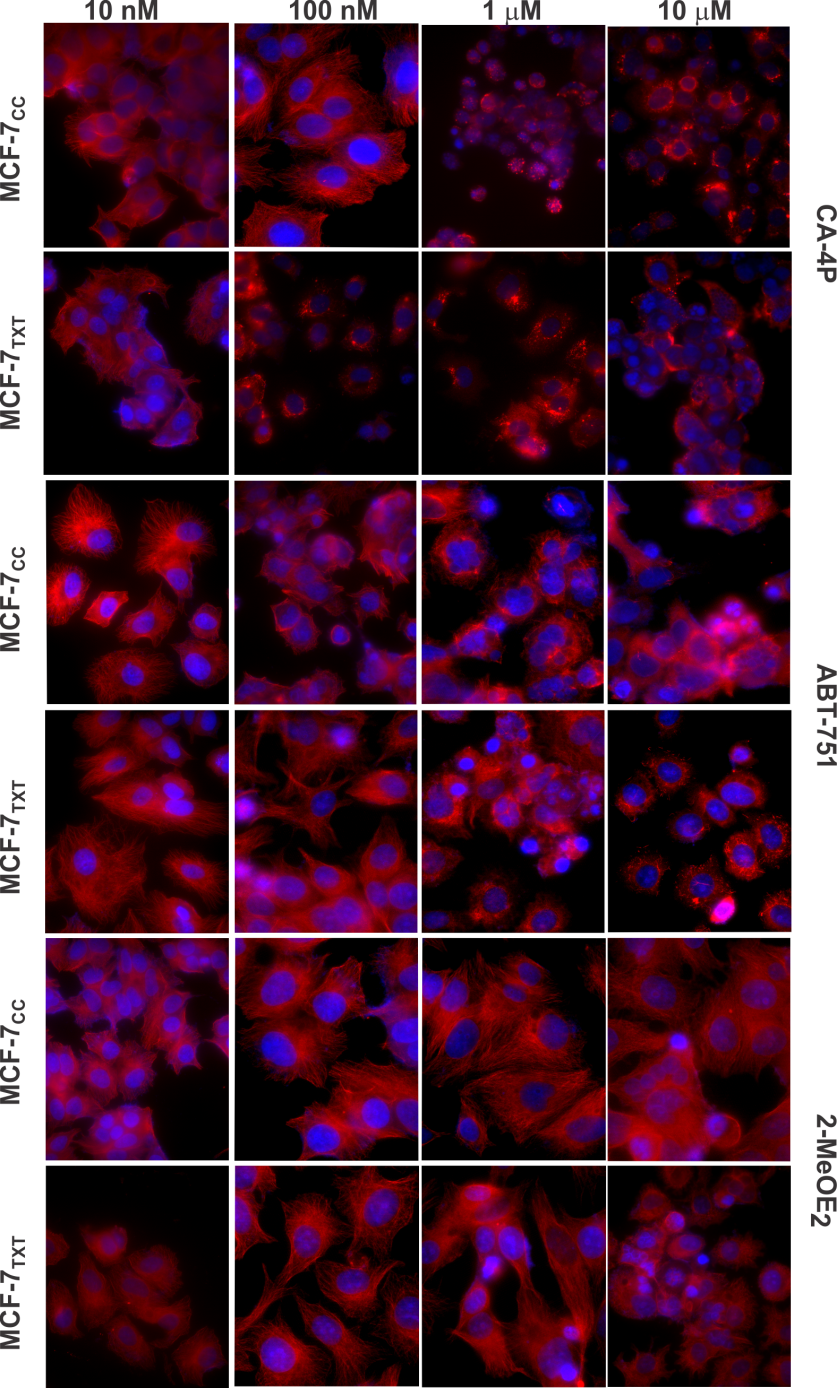
The effects of CA-4P, ABT-751, and 2MeOE_2_ on the microtubule formation, M-phase arrest and cell apoptosis in the selected MCF-7 cell lines. MCF-7_CC_ and MCF-7_TXT_ cells were seeded on coverslip and treated with CA-4P, ABT-751, and 2MeOE_2_ of indicated concentrations for 24 h. Microtubule formation, M-phase arrest and cell apoptosis were determined by fluorescence microscopy. The red staining indicates the α-tubulin and the blue is the DAPI stain for DNA. Size bar: 10 μm.

Like other MTAs, CSBAs kill cells by inducing apoptosis through disrupting microtubule dynamics and arresting cells in mitotsis. We, therefore, examined the effects of these three CSBAs on chromatin morphology and microtubule formation by fluorescence microscopy microtubule formation using the red tubulin stain (Fig 9). Chromatin morphology was revealed by DAPI stain (blue). CA-4P at 100 nM almost completely disrupted microtubule formation in MCF-7_TXT_ cells such that no microtubule fibers visible and all of the tubulin was located in small aggregates (Fig 9A). However, for MCF-7_CC_ cells, 100 nM CA-4P did not significantly disrupt the microtubules and 1 μM CA-4P was required to completely inhibit microtubule formation (Fig 9A). Much higher concentrations of ABT-571 and 2MeOE_2_ were needed to block the microtubule formation in these two cell lines. While 1 μM of ABT-751 caused visible damage to the microtubule system, only 10 μM of ABT-571 completely blocked the formation of microtubule fibres (Fig 9B). For MCF-7_CC_ cells, at 10 μM ABT-571 was still not able to disrupt the microtubule system completely (Fig 9B). The potency of 2MeOE_2_ was even lower than ABT-571: the effects were only visible at 10 μM. The effect of 2MeOE_2_ was stronger on MCF-7_TXT_ cells than MCF-7_CC_ cells (Fig 9C). Similarly, all of these three CSBAs caused fragmentation and other morphology changes in cell chromatin and the effects were stronger in MCF-7_TXT_ cells than in MCF-7_CC_ cells (Fig 9).

In summary, these results further showed that docetaxel-resistant MCF-7_TXT_ cells were more sensitive to CSBAs in terms of cell cytotoxicity, microtubule formation and chromatin fragmentation. Thus CSBAs have the potential to overcome taxane resistance.

## Discussion

The aim of our present research is to identify drugs that could potentially overcome taxane resistance in breast cancer. We previously demonstrated that taxane resistance may be related to the expression of various β-tubulin isoforms and the sensitivity of these β-tubulin isoforms to taxanes in terms of microtubule dynamics [23]. Since taxanes stabilize microtubules, we hypothesized that taxane-resistant breast cancer cells may be more sensitive to microtubule destabilizing agents including vinca alkaloids and CSBAs. To test this hypothesis, we utilized previously established cell lines in which MCF-7 breast cancer cells were selected for survival in increasing concentrations of docetaxel (MCF-7_TXT_ cells) [22]. A cell line selected under identical conditions in the absence of drug (MCF-7_CC_) was used as a control.

Our results indicated that taxane-resistant MCF-7_TXT_ cells were not only cross resistant to vinca alkaloids including vinorelbine and vinblastine, but were even more sensitive to several CSBAs including colchicine, CA-4P, ABT-571 and 2MeOE2. Cytotoxicity assays indicated that the IC_50_ of MCF-7_TXT_ cell to vinorelbine and vinblastine was nearly 10 and 5 times higher, respectively, than that of MCF-7_CC_ cells. However, the IC_50_ of MCF-7_TXT_ cells to colchicine was 5 times lower than that of MCF-7_CC_ cells. Indirect immunofluorescence showed that all MTAs induced the disorganization of microtubules and the chromatin morphology. Interestingly, each MTA caused a unique pattern of microtubule and chromain morphology. Most importantly, MCF-7_TXT_ cells were more resistant to vinorelbine and vinblastine, but more sensitive to colchicine than MCF-7_CC_ cells in terms of microtubule and chromatin morphology. Furthermore, PARP cleavage assays showed that all of these MTAs induced apoptosis of MCF-7 cells. However, MCF-7_TXT_ cells were more resistant to vinorelbine and vinblastine, but more sensitive to colchicine than MCF-7_CC_ cells. Live imaging showed that the microtubule dynamics of MCF-7_TXT_ cells were less sensitive to vinca alkaloids, but more sensitive to colchicine. Finally, we demonstrated using cytotoxicity assays and indirect immunfluorensce that MCF-7_TXT_ cells were also more sensitive to other CSBAs including CA-4P, ABT-571, and 2MeOE_2_. However, these CSBAs have different potency in terms of cell cytotoxicity. The IC_50_s of CA-4P and colchicine were much lower than ABT-571 and 2MeOE_2_. The IC_50_ for 2MeOE_2_ is at the μM level, the highest among all CSBAs examined in this investigation. Moreover, both CA-4P and Colchicine showed the highest efficacy in overcoming docetaxel resistance.

Previous research suggests that CSBAs can target two mechanisms underlying taxane resistance: insensitivity to ABCB1 and having different binding preference to various β-tubulin isoforms [28].

Taxanes are microtubule stabilizing drugs and multiple mechanisms are involved in taxane resistance [13, 23]. The most established mechanism for resistance to more than one chemically unrelated class of agents (multidrug resistance) is the overexpression of drug efflux proteins such as the ATP-binding cassette transporters (ABC) [1, 3, 8, 13]. These include ABCB1, ABCC1 and ABCG2. ABCB1 has been particularly implicated in taxane resistance. Although decreasing ABCB1 expression in 60 cell lines increased the sensitivity to paclitaxel [29] the results from clinic studies are controversial. For example, one study shows that increased ABCB1 expression correlates with shortened disease-free survival [30]. We recently demonstrated that ABCB1 expression is much higher in MCF-7TXT cells than that in MCM-7CC cells [23]. The effects of vinca alkaloids are also sensitive to ABCB1 [11, 28, 31–33]. Thus, it is not surprisinng that docetaxel-resistant MCF-7TXT cells were cross-resistant to both vinorelbine and vinblastine. However, the effects of CSBAs are not exported by ABCB1 [11, 28, 34–38], and thus their toxicity should not be affected by the high ABCB1 expression in taxane-resistant breast cancer cells including MCF-7TXT. Indeed, we did not observe cross resistance to any CSBAs examined in this study.

Additional evidence also supports the role of β-tubulin isoform expression and mutations in taxane resistance in breast cancer cells. Taxanes bind to β-tubulin and taxane-resistant cancer cells could have altered expression and function of certain β-tubulin isotypes. This could result from mutations in β-tubulin, and increased microtubule dynamics associated with altered microtubule-associated protein (MAP) expression [3, 8, 13, 39–42]. Several studies indicate that aberrant expression of specific β-tubulin isoforms and MAPs strongly correlates with resistance to microtubule targeting agents in various cancers including breast, ovarian, lung and prostate cancers [13, 18]. Microtubule-targetting drugs suppress spindle microtubule dynamics, thus inhibiting the metaphase to anaphase transition, blocking mitosis and inducing apoptosis [16]. Tubulin dimers, composed of different β-tubulin isotypes, shows different microtubule dynamics [40, 43, 44]. The expression levels of various β-tubulin isotypes affects the composition of tubulin dimer and microtubules. Thus, it is not surpring that the expression levels of various tubulin isotypes are closely related to the resistance to taxanes. Our group previously demonstrated that the microtubule dynamics of docetaxel-resistant MCF-7_TXT_ cells are insensitive to docetaxel treatment [23]. The expression patterns of β-tubulin isotypes in MCF-7_TXT_ cells are also very different from MCF-7_CC_ cells [23]. Indeed, while increased β2-, β3- and β-4 tubulin are associated with taxane resistance, decreased β3-tubulin has been detected in vinca-resistant cell lines, together with increased microtubule stability [24]. β3-tubulin overexpression confers resistance to paclitaxel and vinorelbine, but does not affect the resistance to colchicine-site binding agents [25]. STX140, but not paclitaxel, inhibits mammary tumour initiation and progression in a mouse model [26].

In conclusion, our novel finding that CSBAs are able to overcome taxane resistance presents the opportunity to develop novel chemotherapy for targeting taxane-resistant breast cancer. While colchicine has been investigated as an anti-cancer drug, its therapeutic value is restrained by its low therapeutic index (low afininty/specificity). Thus, future research should focus on the identification of novel CSBAs to overcome taxane resistance in breast and other cancers.

## Supporting information

S1 Video The effects of docetaxel on the microtubule dynamics of MCF-7_CC_ cells. The Live imaging was performed as described in Materials and Methods. Following the transfection of the cells with GFP-tagged α-tubulin for 24 hours, the cells were incubated with docetaxel of 10 μM for 2 hour. The images of microtubule dynamics of MCF-7_CC_ cells were recorded every 3 minutes by live imaging.
(WMV)

S2 Video The effects of docetaxel on the microtubule dynamics of MCF-7_TXT_ cells. The Live imaging was performed as described in Materials and Methods. Following the transfection of the cells with GFP-tagged α-tubulin for 24 hours, the cells were incubated with docetaxel of 10 μM for 2 hour. The images of microtubule dynamics of MCF-7_TXT_ cells were recorded every 3 minutes by live imaging.
(WMV)

S3 Video The effects of vinorelbine on the microtubule dynamics of MCF-7_CC_ cells. The Live imaging was performed as described in Materials and Methods. Following the transfection of the cells with GFP-tagged α-tubulin for 24 hours, the cells were incubated with viborelbine of 10 μM for 2 hour. The images of microtubule dynamics of MCF-7_CC_ cells were recorded every 3 minutes by live imaging.
(WMV)

S4 Video The effects of vinorelbine on the microtubule dynamics of MCF-7_TXT_ cells. The Live imaging was performed as described in Materials and Methods. Following the transfection of the cells with GFP-tagged α-tubulin for 24 hours, the cells were incubated with vinorelbine of 10 μM for 2 hour. The images of microtubule dynamics of MCF-7_TXT_ cells were recorded every 3 minutes by live imaging.
(WMV)

S5 Video The effects of colchicine on the microtubule dynamics of MCF-7_CC_ cells. The Live imaging was performed as described in Materials and Methods. Following the transfection of the cells with GFP-tagged α-tubulin for 24 hours, the cells were incubated with colchicine of 10 μM for 2 hour. The images of microtubule dynamics of MCF-7_CC_ cells were recorded every 3 minutes by live imaging.
(WMV)

S6 Video The effects of colchicine on the microtubule dynamics of MCF-7_TXT_ cells. The Live imaging was performed as described in Materials and Methods. Following the transfection of the cells with GFP-tagged α-tubulin for 24 hours, the cells were incubated with colchicine of 10 μM for 2 hour. The images of microtubule dynamics of MCF-7_TXT_ cells were recorded every 3 minutes by live imaging.
(WMV)

S1 Data Fig 1A data.xlsx. Primary data for Fig 1A.
(XLSX)

S2 Data Fig 1B data.xlsx. Primary data for Fig 1B.
(XLSX)

S3 Data Fig 1C data.xlsx. Primary data for Fig 1C.
(XLSX)

S4 Data Fig 1D data.xlsx. Primary data for Fig 1D.
(XLSX)

S5 Data Fig 5A data.xlsx. Primary data for Fig 5A.
(XLSX)

S6 Data Fig 5B data.xlsx. Primary data for Fig 5B.
(XLSX)

S7 Data Fig 5C data.xlsx. Primary data for Fig 5C.
(XLSX)

S8 Data Fig 5D data.xlsx. Primary data for Fig 5D.
(XLSX)

S9 Data Fig 6 colchicine data.xlsx. Primary data for Fig 6 colchicine.
(XLSX)

S10 Data Fig 6 docetaxel data.xlsx. Primary data for Fig 6 docetaxel.
(XLSX)

S11 Data Fig 6 vinorelbine data.xlsx. Primary data for Fig 6 viborelbine.
(XLSX)

S12 Data Fig 6 vinblastine data.xlsx. Primary data for Fig 6 vinblastin.
(XLSX)

S13 Data Fig 8A data.xlsx. Primary data for Fig 8A.
(XLSX)

S14 Data Fig 8B data.xlsx. Primary data for Fig 8B.
(XLSX)

S15 Data Fig 8C data.xlsx. Primary data for Fig 8C.
(XLSX)
